# Materials informatics approach to understand aluminum alloys

**DOI:** 10.1080/14686996.2020.1791676

**Published:** 2020-07-29

**Authors:** Ryo Tamura, Makoto Watanabe, Hiroaki Mamiya, Kota Washio, Masao Yano, Katsunori Danno, Akira Kato, Tetsuya Shoji

**Affiliations:** a International Center for Materials Nanoarchitectonics, National Institute for Materials Science, Tsukuba, Japan; b Research and Services Division of Materials Data and Integrated System, National Institute for Materials Science, Tsukuba, Japan; c Research Center for Structural Materials, National Institute for Materials Science, Tsukuba, Japan; d Research Center for Advanced Measurement and Characterization, National Institute for Materials Science, Tsukuba, Japan; e Higashifuji Technical Center, Toyota Motor Corporation, Shizuoka, Japan

**Keywords:** Materials informatics, aluminum alloys, Markov chain Monte Carlo, 404 Materials informatics / Genomics, 106 Metallic materials

## Abstract

The relations between the mechanical properties, heat treatment, and compositions of elements in aluminum alloys are extracted by a materials informatics technique. In our strategy, a machine learning model is first trained by a prepared database to predict the properties of materials. The dependence of the predicted properties on explanatory variables, that is, the type of heat treatment and element composition, is searched using a Markov chain Monte Carlo method. From the dependencies, a factor to obtain the desired properties is investigated. Using targets of 5000, 6000, and 7000 series aluminum alloys, we extracted relations that are difficult to find via simple correlation analysis. Our method is also used to design an experimental plan to optimize the materials properties while promoting the understanding of target materials.

## GRAPHICAL ABSTRACT

**Figure:**
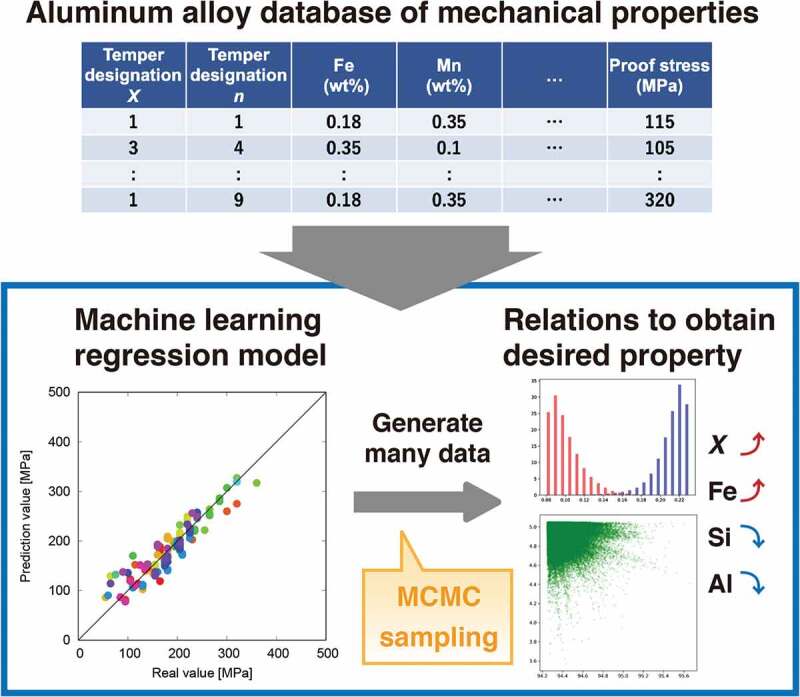

## 1. Introduction

Data-driven techniques are essential in materials science. Applying supervised machine learning to a material’s database can predict the properties of unknown materials from the compositions, structures, and processes without synthesizing the material. As demonstrated in the literature, the predicted properties are helpful for materials discovery [1–14]. On the other hand, it is important to know which compositions, structures, and processes largely affect properties. Hence, one of the original problems in materials science is to extract relations in the databases. This can be tackled by applying correlation analysis [15–18], evaluating the contributions of a prediction model [19–23], and performing a feature selection method [24–29], and applying data-driven techniques to improve the understanding of materials [30].

Recently, the amount of data in public material’s databases based on simulations is increasing. Some databases contain over hundreds of thousands of records [31–33]. Thus, many relations have been elucidated via data-driven techniques applied to materials simulation databases. On the other hand, the results significantly change when focusing on experimental databases. Compared to simulations, experiments in materials science require an enormous amount of time and money, and it is difficult to generate a large amount of experimental data. Furthermore, existing public databases contain little experimental data if the focused property is limited [34]. In many cases, it is difficult to extract relations between properties, compositions, structures, and processes from materials databases based on experiments.

For alloys, calculating the mechanical properties using only simulations is challenging due to the necessity of a macroscopic viewpoint. Consequently, experiments are relied on to determine the mechanical properties in alloys. Because the mechanical properties strongly depend on the dominant element, the number of data in an experimental database for each type of alloy is limited [35]. As a target for data-driven techniques to extract relations, an alloy is one of the representative examples. However, since the dependences of the mechanical properties on processes and element compositions are remarkable, extracting the relations is essential in the development of new alloys. To date, this task has relied on experience and feelings of professional researchers, but alloy development should be accelerated, if such relations are clarified using pure data-driven techniques.

Using data-driven techniques, herein we propose a new strategy to extract relations from alloy experimental databases with small data sizes. First, we train a machine learning prediction model for the mechanical properties when process parameters and compositions of elements are inputted. Here, the prediction model with the highest prediction performance is selected from some supervised machine learning models. Second, the distributions of the process parameters and element compositions based on the prediction model are investigated to obtain the desired mechanical properties. Our strategy employs the Markov chain Monte Carlo (MCMC) method as a generator of new data by the prediction model. Third, we extract factors for the desired mechanical properties, including the influence degree at a glance, to promote the understanding of alloys. Herein we focus on aluminum alloys [36–38] to demonstrate our technique. Our technique finds the relations hidden in the aluminum alloy databases, which are difficult to discover by simple correlation analysis. The most important characteristic of aluminum alloys is their lightness, which contributes to energy-saving practical materials for airplanes and automobiles. These are categorized by some series where the mixed elements differ, and by performing heat treatments, various properties are obtained.

The rest of this paper is organized as follows. Section 2 shows the target aluminum databases for the 5000, 6000, and 7000 series as well as our strategy, which combines a machine learning prediction model and MCMC to search for relations in the database. Section 3 shows the results for the 5000, 6000, and 7000 series using our strategy. We discuss the extracted relations to realize high or low mechanical properties. Section 4 is the discussion and summary.

## 2. Dataset and data analysis method

### 2.1. Aluminum alloys dataset

The target aluminum alloy data are the 5000, 6000, and 7000 series which are summarized in Table 1. The letter in the sample name denotes the alloy shape: (P) plate, (BD) bar-drawn, (T) tube, (BE) bar-extruded, (FH) forging-hand, (S) shape, and (B) brazing. Furthermore, three types of mechanical properties are examined: 0.2% proof stress, tensile strength, and elongation. The data are collected from two databases [39,40]. If an alloy number has multiple data listed, we use the mean value of these data.

**Table 1.** Target aluminum alloys in the 5000, 6000, and 7000 series. Alloy number and temper types are shown.

Sample	Temper		
5000 series (115 data)			
A5005 P	H12, H14, H16, H18, H22, H24, H34, H38		
A5042 P	H11, H18, H19, H24		
A5050 P	H11, H12, H14, H16, H18, H22, H24, H26, H28, H32, H34, H38		
A5052 P	H11, H12, H14, H16, H18, H22, H24, H26, H32, H34, H36, H38		
A5056 BD	H12, H18, H32, H34, H3		
A5082 P	H48		
A5086 P	H11, H18, H22, H24, H26, H32, H34, H36, H38		
A5154 P	H11, H12, H14, H16, H18, H22, H24, H26, H32, H34, H36, H38		
A5182 P	H19		
A5251 T	H11, H12, H14, H16, H18, H22, H24, H26, H32, H34, H36, H38		
A5254 P	H12, H14, H16, H18, H22, H24, H26, H32, H34, H36, H38		
A5454 P	H11, H32, H34		
A5652 P	H11, H12, H14, H16, H18, H22, H24, H26, H32, H34, H36, H38		
A5754 P	H11, H12, H14, H18, H24, H34, H38		
A5N01 P	H12, H14, H16, H32, H34, H36		
6000 series (34 data)			
A6005A BE		T1, T4, T5, T6	
A6005 C BE		T1, T5, T6	
A6060 BE		T4, T5, T6	
A6061 BD		T4, T6	
A6061 P		T4, T6	
A6063 BD		T1, T4, T5, T6	
A6082 P		T4, T6	
A6101 P		T6, T7	
A6151 FH		T6	
A6181 BD		T4, T6	
A6262 BD		T6, T8, T9	
A6463 S		T1, T4, T5, T6	
A6N01 BE		T5, T6	
7000 series (24 data)			
A7072 B			T6
A7003 BE			T5, T6
A7005 S			T5, T6
A7010 P			T6, T7
A7020 BE			T4, T6
A7049A BE			T6, T7
A7050 P			T7
A7075 P			T6, T7
A7178 P			T6, T7
A7204 P			T4, T5, T6
A7475 P			T6, T7
A7N01 P			T4, T5, T6

The 5000 series aluminum alloys are classified into alloys that cannot be heat treated, where Mg is added to increase the strength. In addition to Al (94.25–99.19 wt%) and Mg (0.40–5.05 wt%), Fe (0.13–0.35 wt%), Mn (0.01–0.75 wt%), Si (0.08–0.23 wt%), Ti (0.00–0.10 wt%), Cu (0.02–0.10 wt%), Cr (0.00–0.25 wt%), and Zn (0.015–0.125 wt%) are mixed in the alloys. The two-dimensional temper designations H*Xn* (*X* = 1, 2, 3, 4 and *n* = 1, ..., 9) are used to distinguish the applied combination of basic operations *X* and the degree of strain-hardening *n*. **Table S1** summarizes the meanings of these temper designations [41,42]. The temper designations are used for explanatory variables as well as for the compositions of the elements above. When the mechanical properties are predicted, an integer value of the temper designations is adopted.

The 6000 and 7000 series aluminum alloys are the heat-treatable alloys. For these alloys, the temper designation is given as T*X* (*X* = 1, ..., 10) [41,42] (**Table S2**). In the 6000 series, Fe (0.18–0.50 wt%), Mn (0.02–0.70 wt%), Si (0.40–1.00 wt%), Al (96.16–98.63 wt%), Mg (0.48–1.00 wt%), Ti (0.00–0.08 wt%), Cu (0.05–0.43 wt%), Cr (0.00–0.25 wt%), and Zn (0.05–0.13 wt%) elements are contained, while Fe (0.06–0.35 wt%), Mn (0.03–0.45 wt%), Si (0.05–0.35 wt%), Al (87.05–98.10 wt%), Mg (0.05–2.75 wt%), Ti (0.00–0.17 wt%), Cu (0.05–2.30 wt%), Cr (0.00–1.15 wt%), V (0.00–0.05 wt%), Zr (0.00–0.15 wt%), and Zn (1.05–7.80 wt%) elements are mixed in the 7000 series. The one-dimensional integer temper designation is used as an explanatory variable for the 6000 and 7000 series. The detailed databases for the 5000, 6000, and 7000 series are 5000.csv, 6000.csv, and 7000.csv, respectively.

### 2.2. Materials informatics technique

From the explanatory variables (*i.e*., temper designations and element compositions), which are expressed by vector **x**, supervised machine learning models are created to predict the mechanical properties using the dataset $D={\left\{x_{i},\;f_{i}\right\}}_{i=1,\ldots ,\;N}$, which includes *N* training datapoints. Here, **x**_i_ is the *i*th explanatory variables and *f_i_* is its mechanical property. That is, the prediction function of *f*_pred_(**x**), which depends on **x** is trained using *D*. Although many regression models have been proposed to train *f*_pred_(**x**) in the machine learning community, here, six representative types of methods are utilized: linear regression, ridge regression, elastic net regression, support vector regression, random forest regression, and Gaussian process regression. For the first five methods, we use scikit-learn [43], while the COMBO package [44] is used for the Gaussian process regression. The hyperparameters in these methods are determined so that the leave-one-out cross validation error is minimized. In addition, the prediction performances of machine learning models are compared using the root mean square error (RMSE) for the leave-one-out cross validation, and the most reliable method is selected after optimizing the hyperparameters.

**Figure 1.**
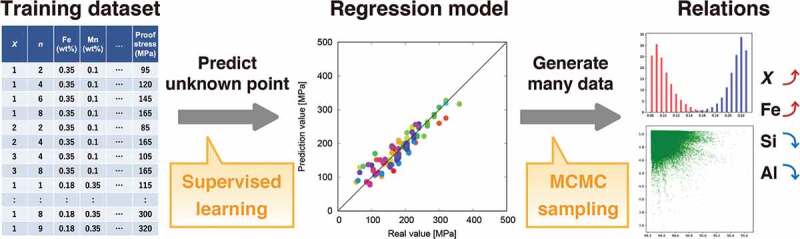
Flow of our strategy to extract the relations by combining a regression model and MCMC.

Our strategy (Figure 1) investigates the distributions of the explanatory variables in the most reliable machine learning model to understand the influence of the explanatory variables and to obtain the desired mechanical properties. We utilize the MCMC method to draw the distribution, which is performed by the emcee package [45,46]. Although this package implements several approaches to update the state, herein the stretch move [47] is adopted. In each Monte Carlo update, all explanatory variables are simultaneously updated. Conventionally, MCMC is used in materials informatics research to analyze the posterior distribution in Bayes statistics [48–50]. Unlike conventional research, MCMC is used as a generator of new data by a prediction model.

We perform MCMC samplings with the probability distribution, *P*(**x**) given in advance. For example, when the probability distribution with positive *T* is set to

(1) $$P\left(x\right)\propto exp\left[-\frac{f_{+}-f_{pred}\left(x\right)}{f_{+}-f_{-}}/T\right],$$

where *f*_+_ and *f*_-_ are the maximum and minimum values in the training dataset ${\left\{f_{i}\right\}}_{i=1,\ldots ,N}$, respectively, explanatory variables with a high predicted mechanical property are actively sampled. Introducing *f*_+_ and *f*_-_ in Eq. (1) means the standardization of mechanical properties. Thus, our strategy can be applied to different target datasets with the common value of *T*. By adopting this probability distribution, the distribution of the explanatory variables to obtain high properties is drawn. On the other hand, explanatory variables with low mechanical properties appear when the probability distribution is set to

(2) $$P\left(x\right)\propto exp\left[-\frac{f_{pred}\left(x\right)-f_{-}}{f_{+}-f_{-}}/T\right],$$

These distributions drawn by MCMC with Eqs. (1) and (2) include information about the values of the temper designations and compositions to obtain higher or lower mechanical properties, respectively.

Furthermore, the width of the histogram calculated by the obtained distributions shows the robustness of each explanatory variable, which should reflect important heat treatments and elements for each mechanical property. Based on such information, the relations between explanatory variables and mechanical properties can be understood quickly via an MCMC-based method. Supplemental note A discusses the probability distribution when the target value of the property is determined in advance. Note that in our implementation of MCMC, the sampling space is limited. It must fall between the minimum and maximum values of the explanatory variables obtained in *D* because predictions by the regression model become meaningless for extrapolated points and extraneous relations will be extracted if this limitation is not imposed. Therefore, in this paper, MCMC draws an interpolation distribution between the minimum and maximum values of the explanatory variables.

Setting the value of *T* is important to obtain reliable relations because the drawn distributions easily fluctuate by changing *T*. To determine the appropriate value of *T* to properly extract relations, we address the dependency of the distributions on *T* by targeting the proof stress and elongation for the 5000 series (**Figure S1)**. If the value of *T* is sufficiently large, an almost random walk is performed, and the distributions for high or low mechanical properties do not differ from using Eqs. (1) and (2). For a random walk, the distributions against each explanatory variable by the emcee package have a higher probability at the center and a lower one at the edges (**Figure S2**). To clearly capture relations from the distributions, we conclude that $T=e^{-5}$ is an appropriate value of *T* for the condition in this paper. Note that theoretically, 99.9% of the sampling points are included in the upper 95% or the lower 95% between *f*_+_ and *f*_-_ when Eqs. (1) and (2) are used, respectively.

Since the appropriate value of *T* should depend on motivations, (*i.e*., whether the obtained distributions of the mechanical properties are acceptable), this conclusion is a guide to determine *T*. This ambiguity of *T* means that the absolute evaluation of the robustness for each explanatory variable is difficult via our proposed strategy. On the other hand, the MCMC method should promote visualization of the relations relative to other variables.

## 3. Results by data-driven analysis

We show the results by our data-driven technique for the 5000, 6000, and 7000 series aluminum alloys. Here, the 0.2% proof stress, tensile strength, and elongation are used as mechanical properties of aluminum alloys. Then the results are compared.

### 3.1. 5000 series aluminum alloys

**Figure 2.**
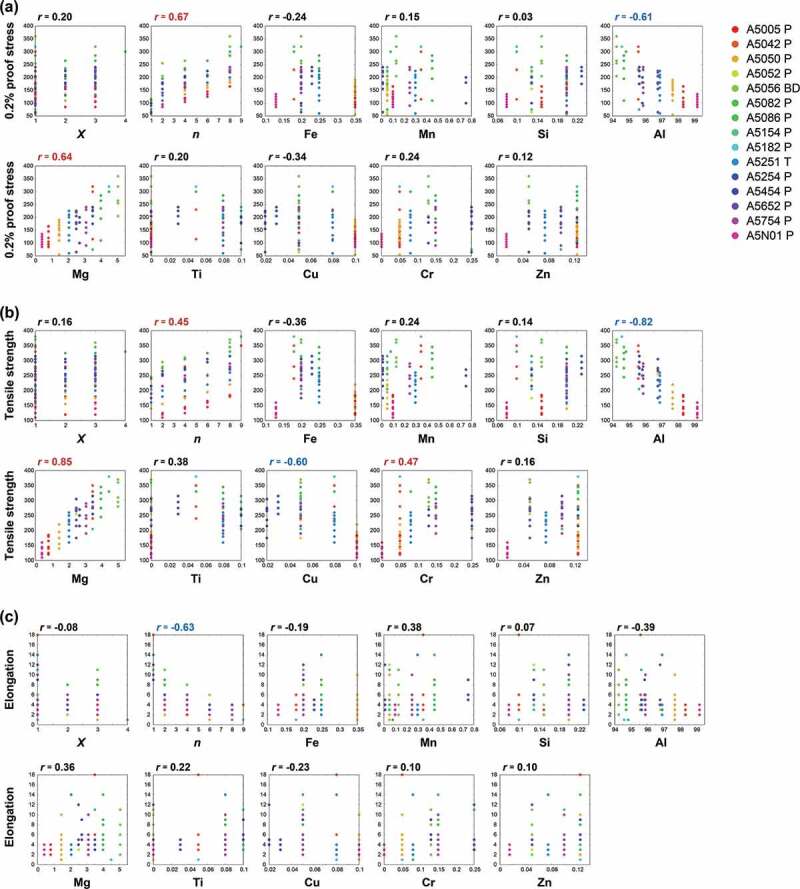
Dependence of the mechanical properties of (a) 0.2% proof stress, (b) tensile strength, and (c) elongation on the temper designations *X* and *n*, and the compositions of nine types of elements in the 5000 series. Values of *r* denote the correlation coefficient. Correlation coefficients in red, blue, and black indicate positive, negative, and no relations, respectively. Each type of aluminum alloy is distinguished by the color of the points.

For the 5000 series, there are 11 dimensions of explanatory variables due to two temper designations *X* and *n* and nine elements, and 115 data are collected. To compare our strategy to conventional analysis, we used a simple correlation analysis as the conventional one. Figure 2 shows pair plots between the mechanical properties and the explanatory variables and the values of the Pearson correlation coefficient, *r*, which is a measure of the linear association between two variables [51]. Here, *r* is evaluated by the Python package pandas.DataFrame.corr [52].

According to Guilford’s informal interpretations [53], relations can be categorized depending on the value of *r* as $\left|r\right|=0.9-1.0$ (very high correlation), $\left|r\right|=0.7-0.9$ (high correlation), $\left|r\right|=0.4-0.7$ (moderate correlation), $\left|r\right|=0.2-0.4$ (low correlation), and $\left|r\right|<0.2$ (slight correlation). Herein we assume that the relations exist when $\left|r\right|>0.4$. In Figure 2, correlation coefficients in red, blue, and black indicate positive, negative, and no relations, respectively. From the results of the correlation coefficients, the second temper designation *n* will increases the proof stress and tensile strength while the elongation decreases. Since the second digit of the temper designation stands for the degree of strain-hardening and it is well known that there is a strong correlation between the hardness and strength of aluminum alloys [54], the obtained trends are reasonable.

On the other hand, Figure 2 shows that the very strong positive relations between *n* and tensile strength are limited to each alloy. However, if the value of *r* evaluated by the whole data is not large, the overall relation is judged to be weakly positive. This is because that the values of the tensile strength strongly depend on the alloy type and the data deviates largely. Compared with *n*, the first temper designation *X* has a negligible effect on the mechanical properties. From a viewpoint of element composition, Al and Mg compositions have outstanding relations for the proof stress. The tensile strength is related with the Al, Mg, Cu, and Cr compositions. However, elongation does not have a clear relation with the element composition.

**Figure 3.**
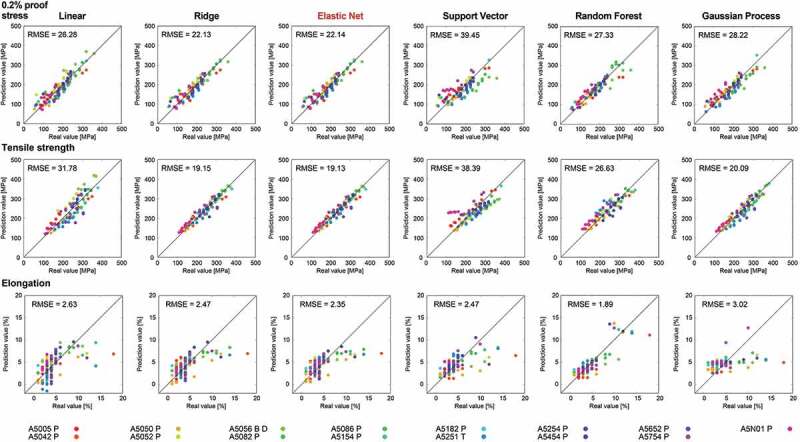
Prediction results by machine learning models for the 0.2% proof stress, tensile strength, and elongation in the 5000 series aluminum alloys. These points are predictions for the test data when the leave-one-out cross validation is performed, that is, for the prediction of each point, target data is not included in the training of the machine learning model. Root mean square error (RMSE) for the test data by the leave-one out method is also denoted. As highlighted in red, the elastic net regression provides a relatively higher prediction accuracy for the three mechanical properties.

Next, our strategy is applied to the 5000 series aluminum alloys. Machine learning prediction models are constructed to predict the mechanical properties from the explanatory variables. Figure 3 shows the scatter plots between the real and predicted mechanical properties for the test data by various machine learning regression models. Here, the test data is prepared and the prediction results are plotted using the leave-one-out cross validation. The RMSE is evaluated for the test data corresponding to the leave-one-out cross validation error, which is denoted in Figure 3. For the proof stress and tensile strength, the prediction is highly efficient regardless of the machine learning model. On the other hand, the prediction performance for elongation is poor compared with the proof stress and tensile strength. Hence, it is more difficult to predict the elongation than the proof stress or tensile strength in 5000 series aluminum alloys. In particular, in the high elongation region, the prediction becomes worse. Among these machine learning models, the elastic net regression has a relatively higher prediction accuracy for the three mechanical properties.

**Figure 4.**
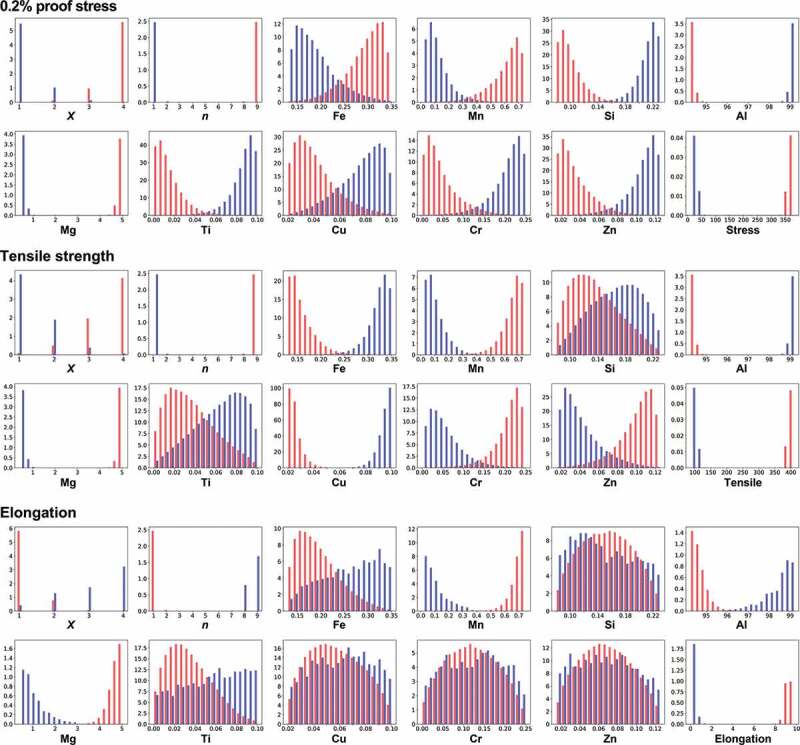
Distributions of temper designations *X* and *n* and compositions of elements to obtain high (red) or low (blue) mechanical properties by MCMC sampling for the 5000 series aluminum alloys. Elastic net regression is used as a machine learning prediction model. Temper designations *X* and *n* have a discrete value, while others have continuous values.

In the second step of our strategy, the distributions of explanatory variables, **x**, by MCMC samplings to obtain the desired properties are investigated based on the reliable trained machine learning model. Figure 4 shows the normalized frequency histograms obtained by MCMC sampling as explained in Sec. 2.2, where the number of sampling points is about 170,000. The vertical axis means the frequencies. Here, the cases where high or low mechanical properties are desired, and Eqs. (1) and (2) are used as the probability distributions in MCMC, respectively. The proof stress remarkably depends on the temper designation *n*, Al, and Mg compositions. For a high proof stress, a smaller Al composition around 95 wt% but a larger Mg composition around 5 wt% are desirable for high temper designation *n*. The opposite conditions are necessary for a low proof stress. Furthermore, all the remaining explanatory variables are slightly related to the proof stress. To increase the proof stress, a large temper designation *X*, higher compositions of Fe and Mn, but lower compositions of Si, Ti, Cu, Cr, and Zn are preferred. Except for *n*, Al, and Mg, these relations are not extracted from the simple correlation analysis shown in Figure 2, demonstrating a benefit of our proposed analysis.

Next, we focus on the tensile strength. Temper designation *n* and the correlations of Mn, Al, Mg, and Cu for the tensile strength exhibit similar behaviors as those for the proof stress. On the other hand, Fe, Cr, and Zn compositions show the opposite behaviors. Unlike in the proof stress, Si and Ti compositions are not strongly related with the tensile strength because the histogram is widespread and their distributions overlap in a wide range. Although both the proof stress and tensile strength are related to material strength, the extracted relations against the element compositions differ slightly.

Finally, we consider the elongation. The behaviors differ drastically from the above properties. The distributions against temper designations show the opposite behaviors as those for the proof stress and tensile strength. Generally, there is a common trend in metals where the elongation decreases as the tensile strength increases [55,56]. Our analysis derives the same trend from the data of aluminum alloys. It also implies that it is difficult to improve these three properties simultaneously. On the other hand, slight relations are observed for the Mn, Al, and Mg compositions, similar to the behaviors for the proof stress and tensile strength.

To increase the three mechanical properties simultaneously, the compositions of Mn and Mg should be increased, while decreasing the Al composition. If scatterplots are drawn between these element compositions of the MCMC sampling points, the relations between explanatory variables and whether simultaneous change of these variables is needed to obtain three high mechanical properties can be visualized. **Figure S3** shows the scatterplots for the Mn, Al, and Mg compositions. Simultaneously changing all three compositions is important in this case. The other element compositions are not strongly related with the elongation, suggesting that it is difficult to correlate these element compositions directly to elongation in aluminum alloys. Table 2 summarizes these relations between the three mechanical properties and each explanatory variable.

**Table 2.** Extracted relations between mechanical properties and each explanatory variable for the 5000 series. White and black triangles denote whether to increase or decrease for high (upper table) or low (lower table) mechanical properties, respectively. Bar indicates almost no relation.

High	*X*	*n*	Fe	Mn	Si	Al	Mg	Ti	Cu	Cr	Zn
0.2% proof stress	Δ	Δ	Δ	Δ	▲	▲	Δ	▲	▲	▲	▲
Tensile strength	Δ	Δ	▲	Δ	-	▲	Δ	-	▲	Δ	Δ
Elongation	▲	▲	-	Δ	-	▲	Δ	-	-	-	-
Low	*X*	*n*	Fe	Mn	Si	Al	Mg	Ti	Cu	Cr	Zn
0.2% proof stress	▲	▲	▲	▲	Δ	Δ	▲	Δ	Δ	Δ	Δ
Tensile strength	▲	▲	Δ	▲	-	Δ	▲	-	Δ	▲	▲
Elongation	Δ	Δ	-	▲	-	Δ	▲	-	-	-	-

The elastic net regression is based on the linear regression, and the coefficients for each explanatory variable can be addressed. MCMC confirms that the distributions are directly reflected by these coefficients. In particular, the coefficients for Si, Cr, and Zn compositions are zero for the elongation in the regression model. For such cases, MCMC performs a random walk in the limited space between the minimum and maximum values of these compositions. In fact, the distributions of Si, Cr, and Zn are similar to the distribution by a random walk (**Figure S2**). On the other hand, our strategy shows a true effect for the regression model where coefficients cannot be easily understood, as addressed in Sec. 3.3.

## 3.2. 6000 series aluminum alloys

For the 6000 series, there are 10 dimensions of explanatory variables because there is one temper designation *X* and nine elements, and 34 data are collected. **Figure S4** shows the pair plot between mechanical properties and explanatory variables. The machine learning prediction results (**Figure S5**) show that the random forest regression has the highest prediction performance for the three mechanical properties.

**Figure 5.**
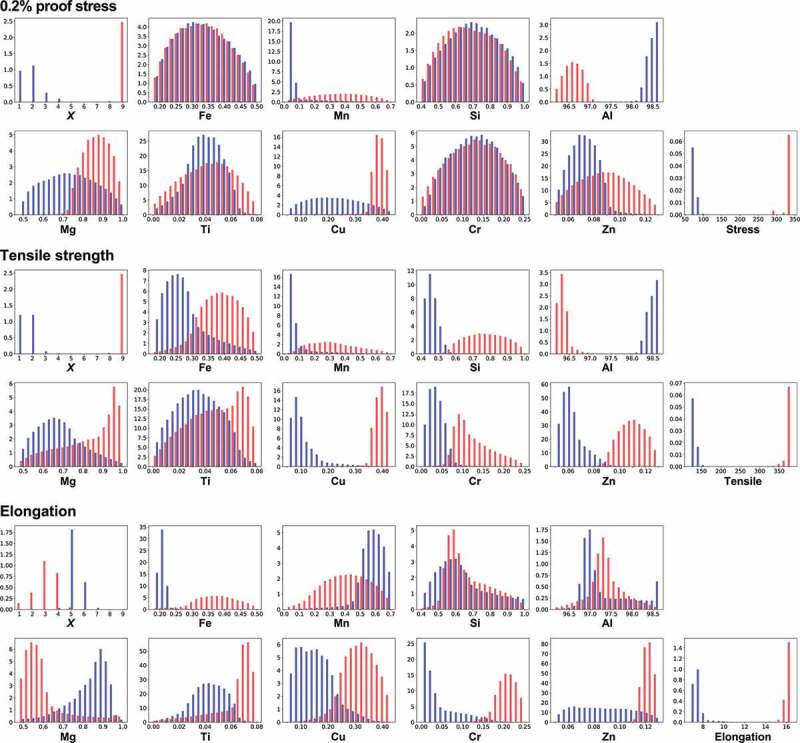
Distributions of temper designation *X* and compositions of elements to obtain high (red) or low (blue) mechanical properties by MCMC sampling in the 6000 series aluminum alloys. Random forest regression is used as a machine learning prediction model. Temper designation *X* has a discrete value, while others have continuous values.

Based on the random forest regression, the frequency histogram is calculated by MCMC sampling (Figure 5). Table 3 summarizes the extracted relations between the three mechanical properties and each explanatory variable from the frequency histograms. The proof stress is remarkably dependent on the temper designation *X* and Al composition to obtain high or low values, but the others do not exhibit strong relations for the proof stress. In particular, the distributions for high and low values nearly overlap for Fe, Ti, Si, and Cr. These distributions are similar to those obtained by a random walk (see **Figure S2**), indicating that these elements are not related to the proof stress. For the tensile strength, many parameters except for the Fe, Mg, and Ti compositions are related when the tensile strength decreases. On the other hand, a large temper designation *X* is preferred along with a low Al and high Cu and Zn compositions to increase the tensile strength. In addition, an optimum value for the Cr composition exists around 0.1 wt%.

**Table 3.** Extracted relations between mechanical properties and each explanatory variable for the 6000 series. White and black triangles denote whether to increase and decrease for high (upper table) or low (lower table) mechanical properties, respectively. Bar indicates almost no relation, while asterisk denotes an optimum value exists.

High	*X*	Fe	Mn	Si	Al	Mg	Ti	Cu	Cr	Zn
0.2% proof stress	Δ	-	-	-	▲	Δ	-	Δ	-	-
Tensile strength	Δ	-	-	-	▲	-	-	Δ	*	Δ
Elongation	*	*	-	*	*	Δ	Δ	Δ	Δ	Δ
Low	*X*	Fe	Mn	Si	Al	Mg	Ti	Cu	Cr	Zn
0.2% proof stress	▲	-	▲	-	Δ	-	-	-	-	▲
Tensile strength	▲	-	▲	▲	Δ	-	-	▲	▲	▲
Elongation	*	▲	Δ	-	*	Δ	-	▲	▲	-

As an interesting relation which can be revealed by our strategy, there is a relation in the lower property region, but in the higher property region there is no relation against the explanatory variable or vice versa. One such example is the Mn composition for the proof stress and tensile strength. For such cases, a simple correlation analysis is not useful as it will indicate weak relations exist in the whole region. This means that our strategy can capture the nonlinear relations between the mechanical properties and explanatory variables. This is a clear advantage compared with simple correlation analysis.

Furthermore, to realize a high elongation, the Ti, Cu, Cr, and Zn compositions should be increased when *X* is fixed to 3 or 4. Conversely, to decrease elongation, the Fe, Cu, and Cr compositions should be decreased while the Mn and Mg compositions are increased when *X* is fixed to 5. Note that our strategy can extract a lot of relations for the elongation compared to a simple correlation analysis (**Figure S4**). If three high mechanical properties are desired, then Cu should be increased as it is the only common relation for the three properties.

## 3.3. 7000 series aluminum alloys

For the 7000 series, there are 12 dimensions of explanatory variables because there is one temper designation *X* and eleven elements, and 24 data are collected. **Figures S6** and **S7** show the pair plots and machine learning prediction results, respectively. The support vector regression has the highest prediction performance for the three mechanical properties. However, the prediction performances are worse than those for the 5000 and 6000 series. In particular, the prediction performance for elongation is insufficient.

**Figure 6.**
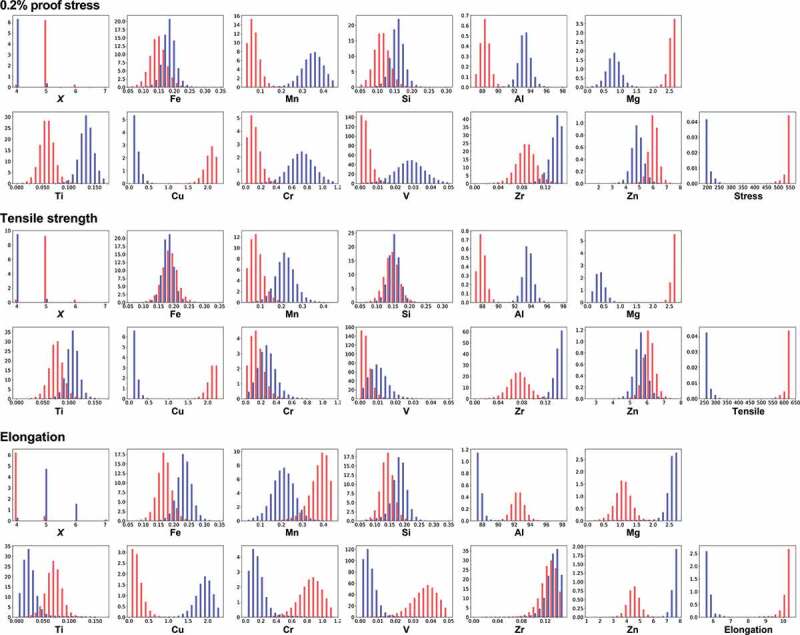
Distributions of temper designation *X* and compositions of elements to obtain high (red) or low (blue) mechanical properties by MCMC sampling in the 7000 series. Support vector regression is used as a machine learning prediction model. Temper designation *X* has a discrete value, while others have continuous values.

Based on the support vector regression, the frequency histogram is calculated by MCMC sampling (Figure 6). Table 4 summarizes the extracted relations between the mechanical properties and each explanatory variable from the frequency histogram. In many cases, an optimum value exists to obtain high or low mechanical properties, which cannot be understood via simple correlation analysis using correlation coefficients. For a high proof stress and a tensile strength, the relations are the same. That is, the compositions of Mg and Cu should be increased, while the Mn, Al, Cr, and V compositions are decreased. On the other hand, almost the opposite relations are observed for high elongation, demonstrating that it is difficult to obtain alloys with the three high mechanical properties in the 7000 series.

**Table 4.** Extracted relations between the mechanical properties and each explanatory variable for the 7000 series. White and black triangles denote whether to increase and decrease for high (upper table) or low (lower table) mechanical properties, respectively. Bar indicates almost no relation, while asterisk denotes an optimum value exists.

High	*X*	Fe	Mn	Si	Al	Mg	Ti	Cu	Cr	V	Zr	Zn
0.2% proof stress	*	*	▲	*	▲	Δ	*	Δ	▲	▲	*	*
Tensile strength	*	-	▲	-	▲	Δ	*	Δ	▲	▲	*	-
Elongation	▲	*	Δ	*	*	*	*	▲	Δ	Δ	Δ	*
Low	*X*	Fe	Mn	Si	Al	Mg	Ti	Cu	Cr	V	Zr	Zn
0.2% proof stress	▲	*	▲	*	*	*	Δ	▲	*	*	Δ	*
Tensile strength	▲	-	*	-	*	▲	*	▲	▲	▲	Δ	-
Elongation	*	*	*	*	▲	Δ	▲	Δ	▲	▲	Δ	Δ

## 4. Discussion and summary

We have proposed a materials informatics technique that combines a machine learning prediction model and MCMC to extract the relations in materials databases. Our strategy can visually understand relations between the properties of materials and explanatory variables such as the compositions of elements, structures, and processes to obtain the desired properties. Our implementation of the strategy is available on GitHub [57]. Herein we target aluminum alloys of 5000, 6000, and 7000 series and show that relations, which are difficult to understand using simple correlation analysis, can be extracted. For example, in the 5000 series, aluminum alloys with high proof stress, tensile strength, and elongation may be realized by increasing the Mn and Mg compositions and decreasing the Al composition. In the 6000 series, temper designation *X* and Al composition are predominantly related to the proof stress and tensile strength, while the others have small relations. Furthermore, if three high mechanical properties are desired, the Cu composition should be large. For the 7000 series, almost the opposite relations are observed between the proof stress/tensile strength and elongation against the compositions of elements. Thus, it is difficult to optimize the mechanical properties simultaneously. Such information is useful to develop new aluminum alloys that meet the needs of industry.

It should be noted that these extracted relations are for the prepared dataset. It is possible that the extracted relations will differ if the dataset is expanded. Thus, to extract reliable relations, a certain amount of reliable data is required so that a machine learning prediction model with sufficient prediction accuracy can be trained. If the prediction accuracy is low, the reliability of the drawn distribution by MCMC will be poor. Accordingly, the reliability of the extracted relations will be low. For example, in our demonstration, the prediction accuracy of the regression model used in the 7000 series is insufficient, and the reliability of the extracted relations is not high. Therefore, to extract reliable relations by our strategy, we should prepare a certain amount of reliable data.

The main advantage of our proposed strategy is that it can be applied to all regression methods to extract relations in the dataset. Conventionally, regression methods (*e.g*., linear and random forest regressions) are limited when attempting to obtain the contributions of each explanatory variable in the prediction model. In contrast, our strategy can obtain the dependencies of the predicted properties on explanatory variables for every regression method. Compared with conventional techniques, our strategy is universal and can extract the relations without limitations due to the machine learning model.

Moreover, our technique can be used to design an experimental plan to optimize the properties while promoting the understanding of target materials. That is, our strategy can be used to find the direction of the explanatory variables which will realize the desired mechanical properties. This can work not only for a single target property but also for multiple target properties with completely positive relations. On the other hand, if we want to improve some properties with opposite relations (*e.g*., the proof stress and elongation in the 7000 series) simultaneously, it is difficult to determine the appropriate setting of the next experiment. However, through our data-driven analysis, such difficulties in the experimental design can also be understood. Since our technique has diverse uses, we believe that it is a valuable tool to develop new materials, including alloys.

## Supplementary Material

Supplemental Material
