# Supplementary material for: Materials informatics approach to understand aluminum alloys
Source: Sci Technol Adv Mater. 2020 Jul 29;21(1):540–51. doi: 10.1080/14686996.2020.1791676 (PMC7476514; doi:10.1080/14686996.2020.1791676)
Supplement: Supplemental Material [file TSTA_A_1791676_SM4022.pdf]

Supplementary information for

Materials informatics approach to understand aluminum alloys

Ryo Tamura*[1,2], Makoto Watanabe[3], Hiroaki Mamiya[4], Kota Washio[5], Masao Yano[5], Katsunori Danno[5], Akira Kato[5], and Tetsuya Shoji[5]

[1]International Center for Materials Nanoarchitectonics, National Institute for Materials Science, Tsukuba 305-0044, Japan

[2]Research and Services Division of Materials Data and Integrated System, National Institute for Materials Science, Tsukuba 305-0047, Japan

[3]Research Center for Structural Materials, National Institute for Materials Science, Tsukuba 305-0047, Japan

[4]Research Center for Advanced Measurement and Characterization, National Institute for Materials Science, Tsukuba 305-0044, Japan

[5]Higashifuji Technical Center, Toyota Motor Corporation, Shizuoka 410-1193, Japan

Table S1. Meanings of temper designations X (left) and n (right) in HXn for the 5000 series[41,42].

X	Means
H1n	Strain hardened without a thermal treatment
H2n	Strain hardened and partially annealed
H3n	Strain hardened and stabilized
H4n	Strain hardened and painted

n	Means
HX1	1/8 hard
HX2	1/4 hard
HX3	3/8 hard
HX4	1/2 hard
HX5	5/8 hard
HX6	3/4 hard
HX7	7/8 hard
HX8	Full hard
HX9	Extra hard

Table S2. Meanings of temper designation X in TX for the 6000 and 7000 series[41,42].

X	Means
T1	Cooled from hot working and naturally aged
T2	Cooled from hot working, cold-worked, and naturally aged
T3	Solution heat treated and cold worked
T4	Solution heat treated and naturally aged
T5	Cooled from hot working and artificially aged
T6	Solution heat treated and artificially aged
T7	Solution heat treated and stabilized
T8	Solution heat treated, cold worked, and artificially aged
T9	Solution heat treated, artificially aged, and cold worked

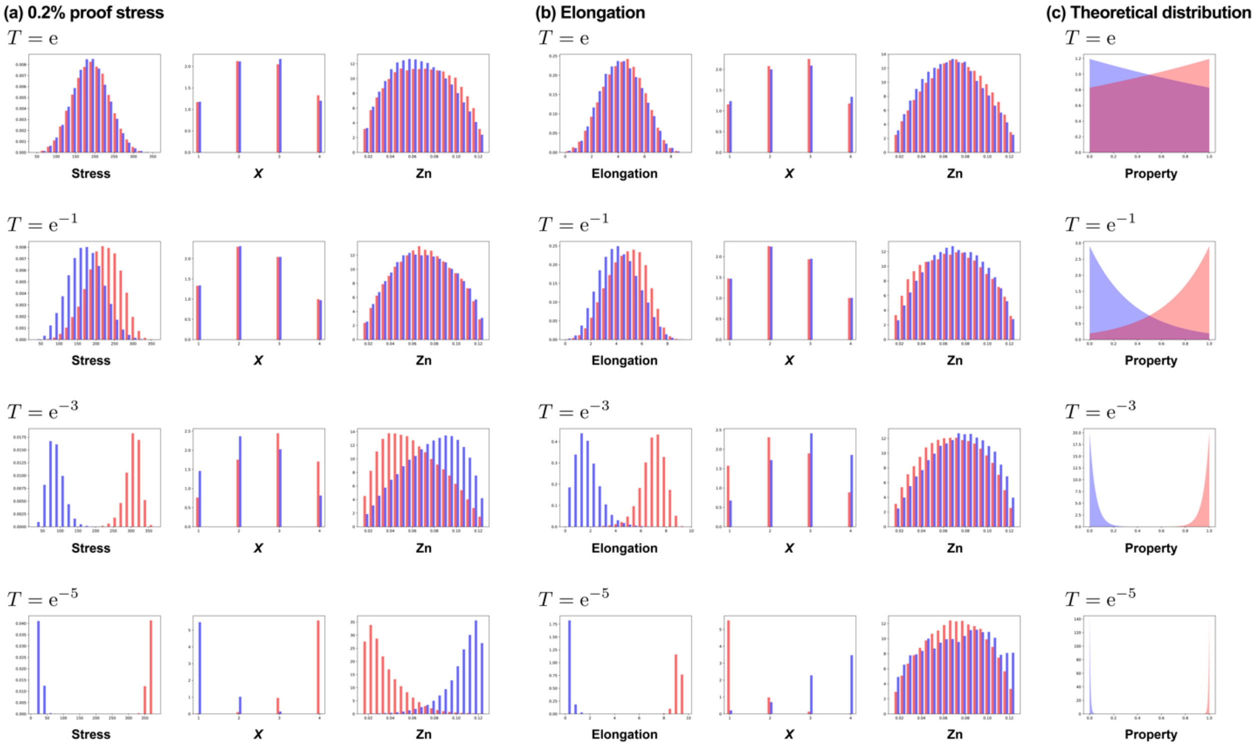

Figure S1. Distributions dependence on T when (a) proof stress and (b) elongation for the 5000 series are targeted. As an example, the distributions of the mechanical property, temper designation X, and Zn composition are shown. With decreasing T, the dependency of the explanatory variables becomes clear except for the Zn composition in the elongation where the coefficient in the regression model for Zn is zero. Theoretical probability distribution depending on T is shown in (c) when $f_+ = 1$ and $f_- = 0$. As T decreases, the probability increases around the high or low properties. In the theoretical distribution, 5.9% ($T = \mathrm{e}$), 13.6% ($T = \mathrm{e}^{-1}$), 63.4% ($T = \mathrm{e}^{-3}$), and 99.9% ($T = \mathrm{e}^{-5}$) sampling points are included in upper 95% or lower 95%, respectively. On the other hand, if T decreases from $T = \mathrm{e}^{-5}$, the MCMC sampling is frozen at the initial state. To clearly extract the relations, we conclude that $T = \mathrm{e}^{-5}$ is an appropriate value of T in this paper.

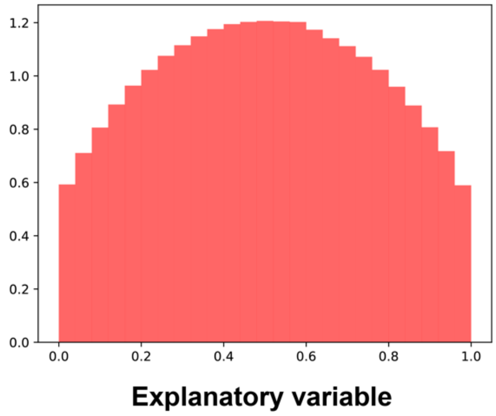

Figure S2. Distribution in a random walk by the emcee package with stretch move. Sampling space is a one-dimensional space limited between 0 and 1.

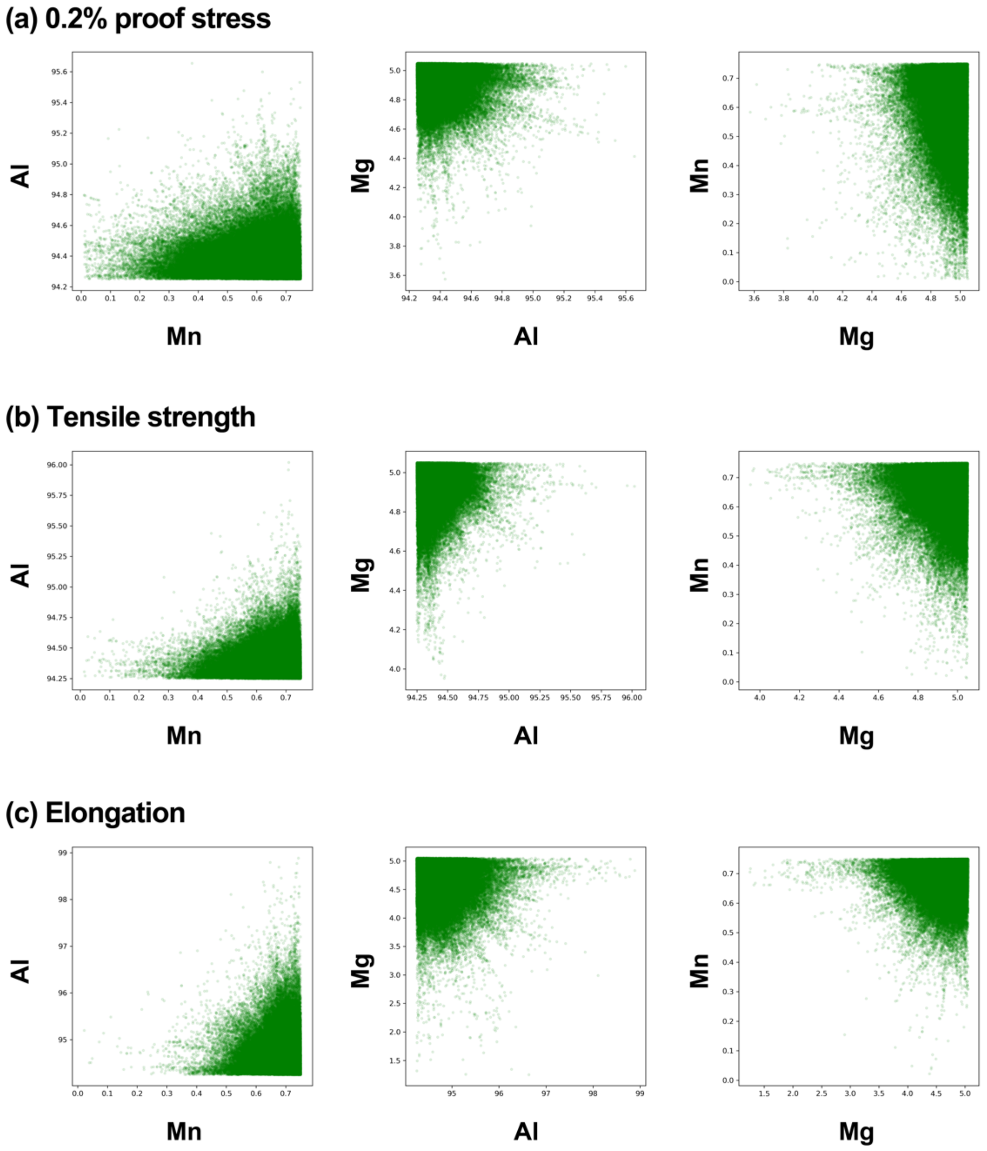

Figure S3. Scatterplots between the Mn, Al, and Mg compositions of MCMC sampling points to obtain large (a) proof stress, (b) tensile strength, and (c) elongation in the 5000 series. In the region where Mn and Mg are larger but Al is smaller, the sampling points are crowded. This indicates that a simultaneous change in these element compositions is important to obtain large three mechanical properties.

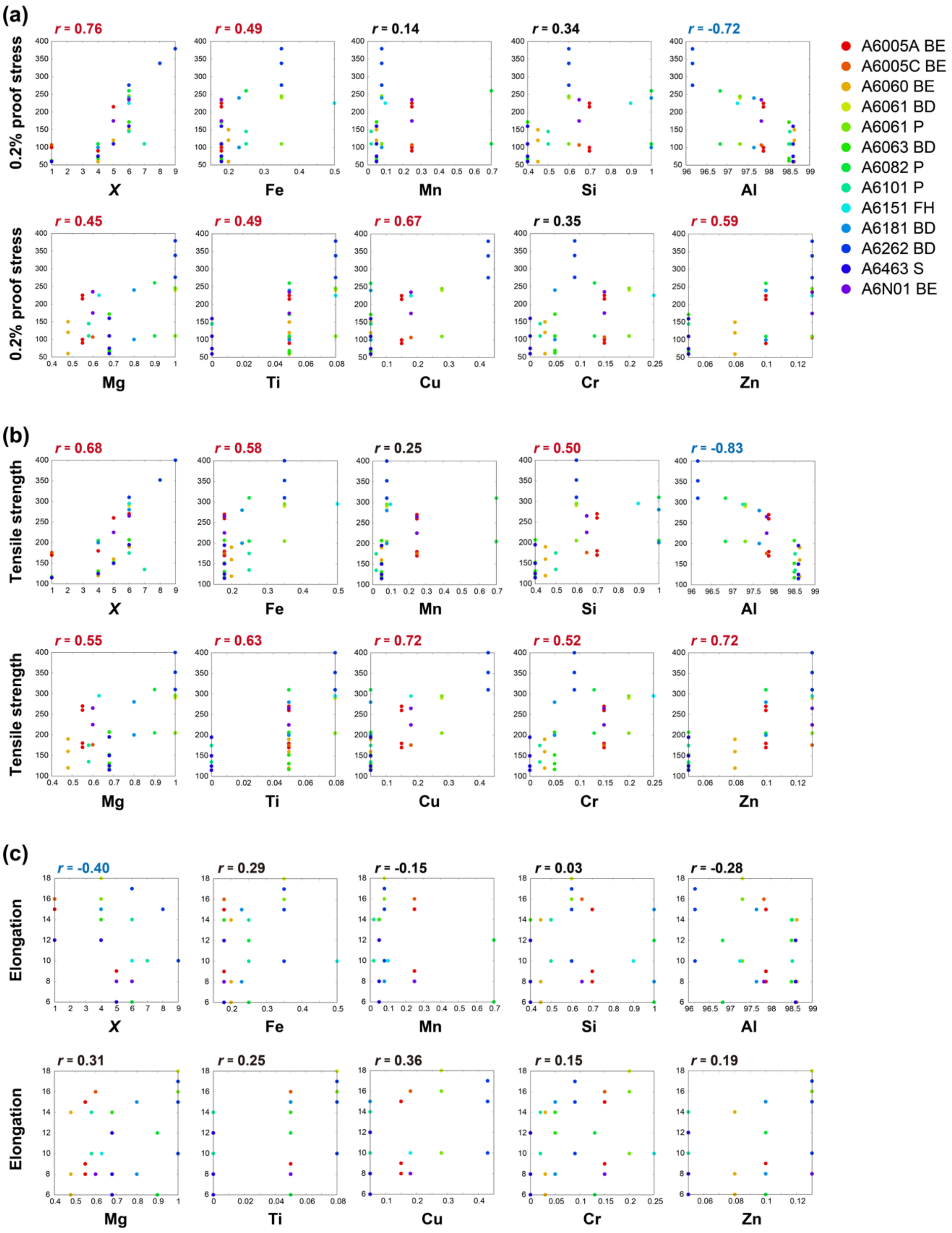

Figure S4. Dependences of the mechanical properties of (a) 0.2% proof stress, (b) tensile strength, and (c) elongation on the temper designation X and compositions for nine types of elements in the 6000 series. Values of r denote the correlation coefficient.

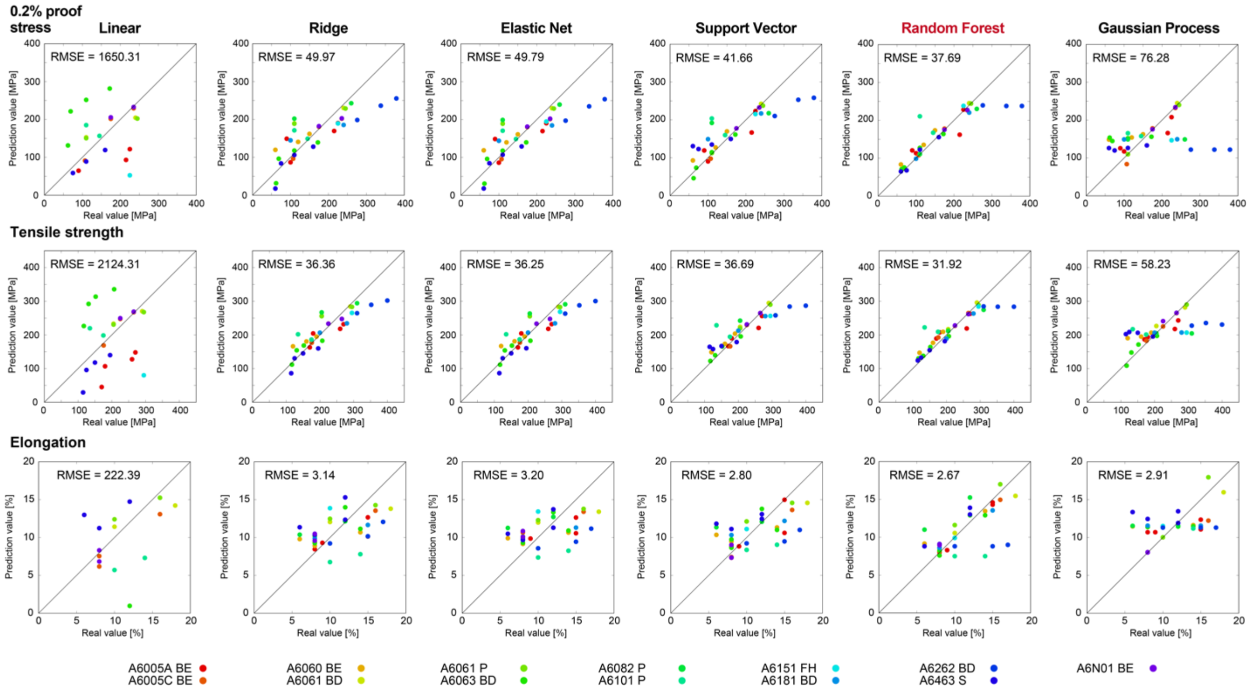

Figure S5. Prediction results by machine learning models for 0.2% proof stress, tensile strength, and elongation in the 6000 series aluminum alloys. These points are predictions for the test data when the leave-one-out cross validation is performed. That is, for the prediction of each point, the target data is not included in the training of the machine learning model. Root mean square error (RMSE) for the test data by the leave-one out method is also denoted. Random forest regression highlighted in red text provides a relatively higher prediction accuracy for the three mechanical properties.

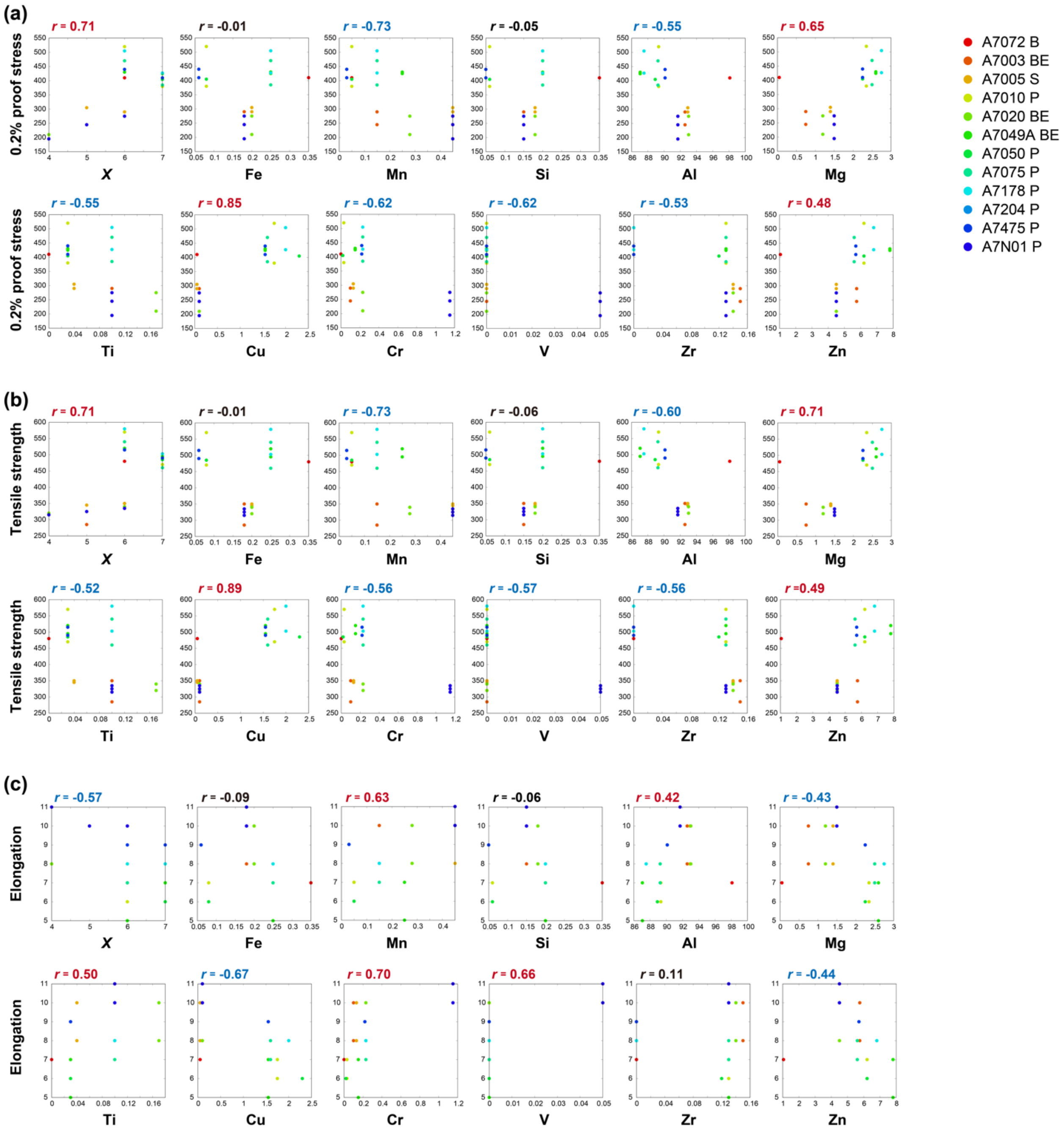

Figure S6. Dependence of the mechanical properties of (a) 0.2% proof stress, (b) tensile strength, and (c) elongation on the temper designation X and compositions of the 11 types of elements in the 7000 series. Values of r denote the correlation coefficient.

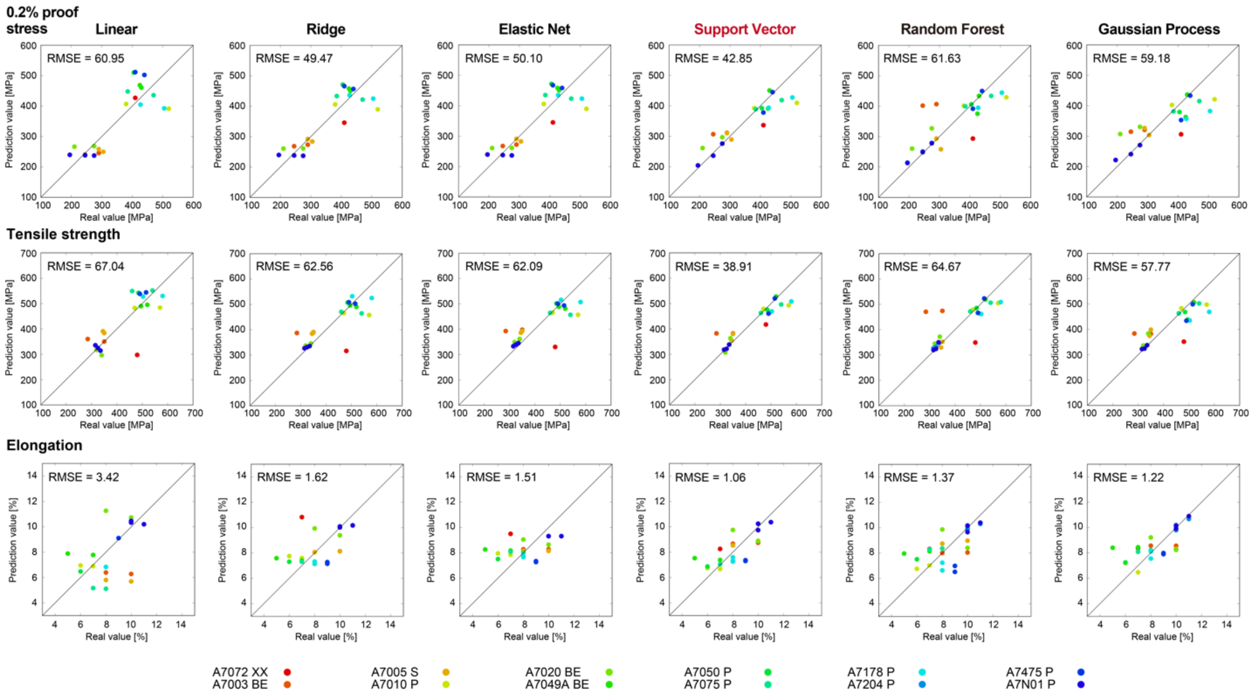

Figure S7. Prediction results by machine learning models for 0.2% proof stress, tensile strength, and elongation in the 7000 series aluminum alloys. These points are predictions for the test data when the leave-one-out cross validation is performed. That is, for the prediction of each point, the target data is not included in the training of the machine learning model. Root mean square error (RMSE) for the test data by the leave-one out method is also denoted. Support vector regression highlighted in red text provides a relatively higher prediction accuracy for the three mechanical properties.

Supplemental Note A: Distributions for the target properties

This supplemental note considers the case where the desired value of the target property is determined in advance. Here, when the target value is given as α, the distributions of the explanatory variables to obtain α can be drawn by setting the probability distribution as

$$P(\mathbf{x}) \propto \exp\left[-\frac{\left(f_{\mathrm{pred}}(\mathbf{x})-\alpha\right)^2}{\max\{(f_{+}-\alpha)^2, (f_{-}-\alpha)^2\}}/T\right], \quad \text{(S1)}$$

with positive T for MCMC. In **Figure S8**, the frequency histograms for the proof stress obtained by MCMC sampling are shown for $T = \mathrm{e}^{-5}$ in the 5000 series aluminum alloys, where α is set to 150 MPa or 250 MPa. Here, the number of sampling points is about 170,000. The optimum values of the temper designation n, Al and Mg compositions are slight for both cases. On the other hand, other explanatory variables are not related to the proof stress because the histogram is widespread and the two distributions overlap in a wide range. In this way, when the target property is determined in advance, our strategy can extract the relation between the materials properties and explanatory variables.

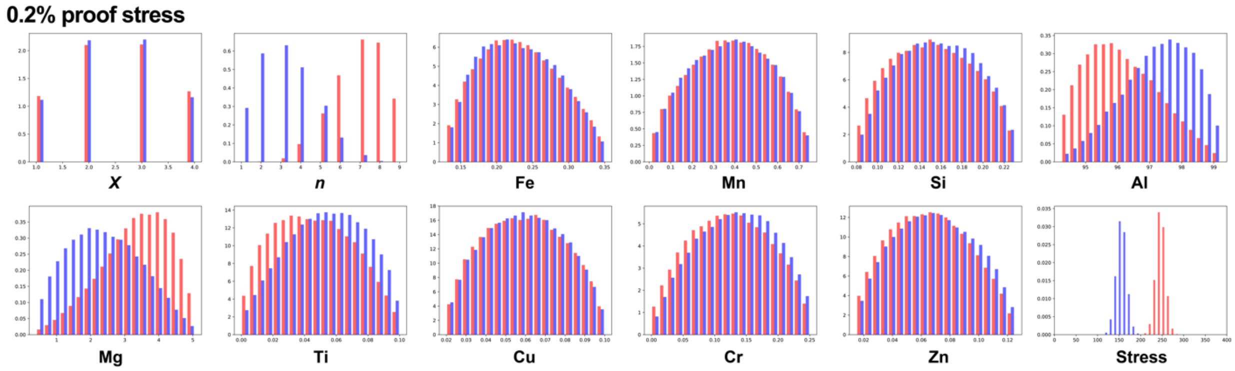

Figure S8. Distributions of temper designations X and n and the compositions of elements to obtain 150 MPa (blue) or 250 MPa (red) of the proof stress by MCMC sampling in the 5000 series aluminum alloys. Elastic net regression is used as a machine learning prediction model. Temper designations X and n have discrete values, while others have continuous values.
